# Systemic absorption and safety of topical terbinafine hydrochloride 10% solution (MOB015B): a phase 1 maximal usage trial in patients with moderate-to-severe onychomycosis

**DOI:** 10.1128/aac.00682-24

**Published:** 2024-08-19

**Authors:** Amir Tavakkol, Janet C. DuBois, Aditya K. Gupta

**Affiliations:** 1 Moberg Pharma AB, Bromma, Sweden; 2 DermResearch Inc., Austin, Texas, USA; 3 Division of Dermatology, Temerty Faculty of Medicine, University of Toronto, Toronto, Ontario, Canada; 4 Mediprobe Research Inc., London, Ontario, Canada; University of Iowa, Iowa City, Iowa, USA

**Keywords:** terbinafine, pharmacokinetics, onychomycosis

## Abstract

Topical antifungals may be considered to treat onychomycosis with minimal risk of systemic side effects. In this study, we assess the safety, tolerability, systemic exposure, and pharmacokinetic characteristics of topical terbinafine hydrochloride 10% solution (MOB015B) in adults with moderate-to-severe onychomycosis. Clinically and mycologically confirmed patients with toenail onychomycosis (*N* = 20) were enrolled in this single-center, open-label study . Each patient had ≥50% involvement of both great toenails and at least four additional toenails affected. MOB015B was applied once daily to all toenails for 28 days. Blood was drawn on days 1, 14, and 28. Plasma concentrations of MOB015B after the first dose were quantifiable in all subjects by 24 h. Steady-state levels in plasma were reached by day 28. The mean systemic exposure on day 28 of 0.72 ng/mL for maximum plasma concentration (C_max_) was approximately 2,000 times lower than the mean plasma level of 1.39 µg/mL seen after oral administration of 250 mg terbinafine for 28 days. Adverse events (five patients), such as headache (*n* = 3), seasonal allergy (*n* = 1), and neck pain (*n* = 1), were considered unrelated to MOB015B; no application site reactions or study discontinuations due to an adverse event were observed. MOB015B applied to all affected toenails under maximal usage conditions for 28 days demonstrated very low levels of terbinafine in plasma (C_max_ <1 ng/mL after 28 days), consistent with a favorable safety and tolerability profile.

**CLINICAL TRIALS:**

This study is registered with ClinicalTrials.gov as NCT03244280.

## INTRODUCTION

Terbinafine is the most prescribed antifungal agent globally and is commonly used as a first-line medication for the treatment of onychomycosis as well as other superficial fungal infections (1–3). Initially introduced in the United States in the 1990s (4, 5), terbinafine inhibits fungal growth by disrupting the sterol biosynthesis pathway. Specifically, it interferes with the formation of ergosterol by inhibiting squalene epoxidase—an enzyme that catalyzes the conversion of squalene to an ergosterol precursor (6). The resultant deficiency in ergosterol compromises cell wall integrity and contributes to impaired growth leading to cell death (6).

When administered orally, terbinafine is primarily absorbed through the gastrointestinal tract at a rapid rate, reaching the peak plasma concentration within 2 h (4), following which, it is distributed readily to the sebum and stratum corneum, resulting in elevated drug levels in the nail plate that exceed plasma levels (6, 7). Before excretion, terbinafine is extensively metabolized by at least seven cytochrome p450 enzymes in the liver; none of the metabolites have demonstrated comparable antifungal activities to terbinafine (4, 8). Although considered an effective treatment option for toenail onychomycosis, with a 70% mycological cure rate and a 38% complete cure rate (negative mycology with normal-appearing nail plate) (4), oral terbinafine can have side effects. Although these are mostly mild in severity and self-resolving, they include smell disturbance, loss of taste, liver enzyme anomalies, and drug interactions (4, 8, 9). Cutaneous adverse events (AEs), such as exfoliative dermatitis and erythema multiforme, and serious AEs, such as fulminant liver failure, have been reported but are rare (9–11). Despite its broad use in patients with mild-to-moderate or severe disease, the option of topical agents with a more favorable safety profile may be warranted for patients on multiple medications, the elderly, or those unwilling to take oral medication. Furthermore, current high rates of onychomycosis recurrence (10%–53%) call for the use of topical agents as prophylaxis (12–14), or use as maintenance therapy once cure has been achieved (15).

MOB015B, a topical terbinafine hydrochloride 10% solution, was developed and evaluated in two phase three trials for the treatment of mild-to-moderate onychomycosis affecting up to 60% of the great toenail (16, 17). Significantly higher mycological cure rates were found in patients treated with MOB015B for 48 weeks compared with the vehicle (69.9% vs. 27.7%) (16) and ciclopirox (80.4% vs. 41.7%) (17); complete cure rate was significantly higher than that of the vehicle and was comparable to that of ciclopirox. In another study, MOB015B was shown to have greater target tissue bioavailability compared with oral terbinafine with significant penetration of the nail plate (1,610 µg/g) and nail bed (45 µg/g) after 24 weeks of treatment (18, 19). The aim of the present study was to assess the systemic absorption pattern, pharmacokinetic (PK) profile, and safety of MOB015B in patients with onychomycosis treated with a maximal-use regimen.

## RESULTS

A total of 20 patients were enrolled in the study. Overall, 85% (17/20) of patients were fully compliant with the dosing regimen and sample collection schedule. One patient (01–017) voluntarily withdrew from the study on day 1 (after a blood sample was taken at 0.5 h) unrelated to any adverse events and was thus excluded from the PK population due to the plasma MOB015B concentrations being below the lower limit of quantification. Minor deviations from the study protocol occurred in two patients – one patient (01–015) missed one dose on day 25, and another patient (01–020) missed two doses on days 26 and 27—and were deemed to have no overall effects on the PK and safety assessments. Baseline characteristics of the study cohort are summarized in Table 1. The PK population consists of 19 patients, and the safety population consists of 20 patients.

**TABLE 1:** Baseline characteristics of enrolled patients (*N* = 20)

Parameter	Value
Age (years), mean (SD)	48.5 (11.4)
Sex, % (n)	
Female	15.0 (3)
Male	85.0 (17)
Race, % (n)	
American Indian or Alaska Native	5.0 (1)
Black or African American	20.0 (4)
White	75.0 (15)
Ethnicity, % (n)	
Hispanic or Latino	45.0 (9)
Not Hispanic or Latino	55.0 (11)
BMI (kg/m^2^), mean (SD)	29.3 (3.8)
Tinea pedis, % (n)	10.0 (2)
Type 2 diabetes mellitus, % (n)	15.0 (3)
Hypercholesterolemia, % (n)	10.0 (2)
Hypertension	15.0 (3)

**Fig 1:**
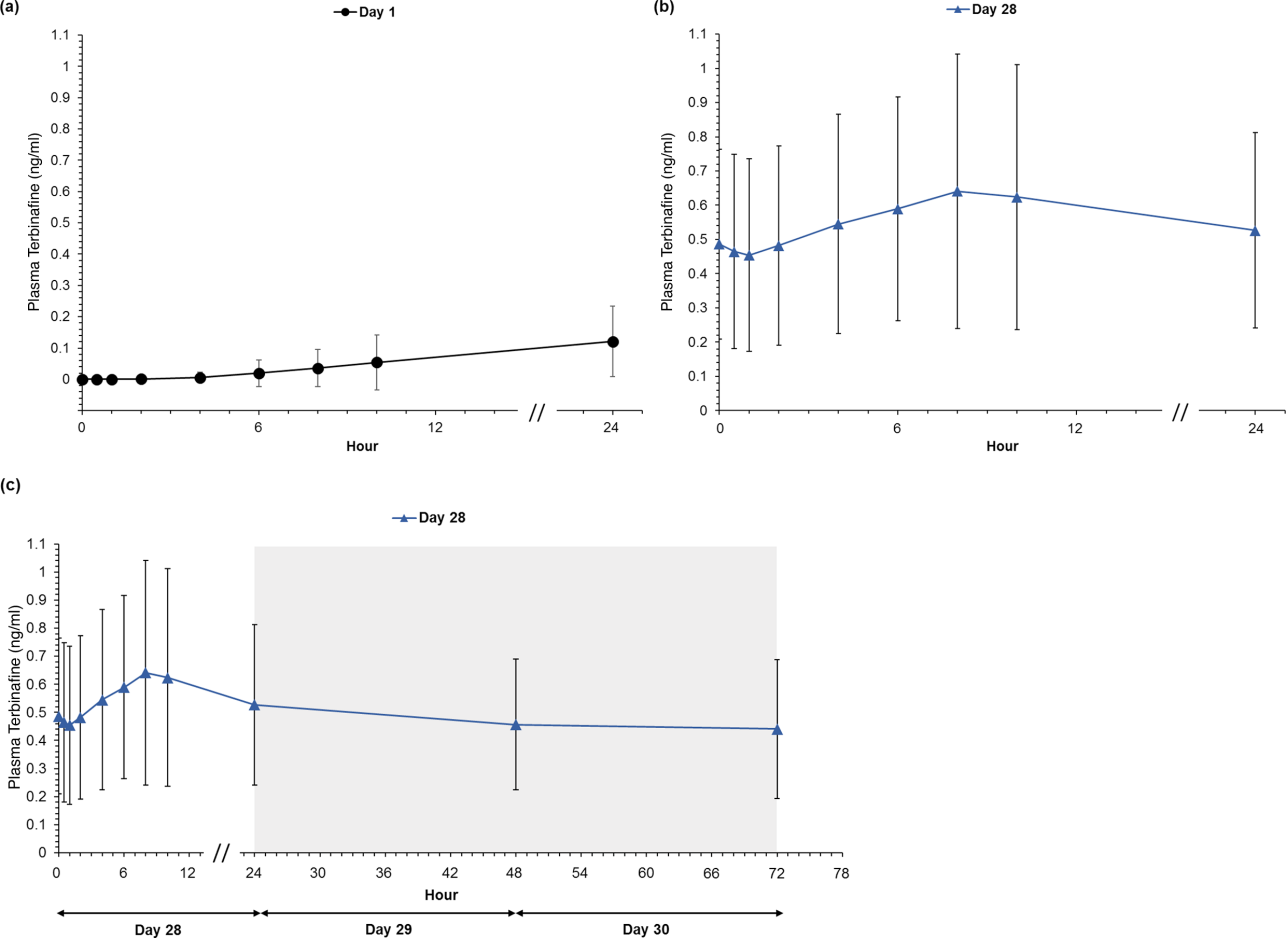
Mean plasma terbinafine concentrations observed in the PK population (*N* = 19) on (**a**) day 1 from 0 to 24 h, (**b**) day 28 from 0 to 24 h, and (**c**) day 28 from 0 h to post-treatment. Gray-shaded area denotes the post-treatment period. All values below the lower limit of quantification (10 pg/mL) were imputed as 0; error bars represent standard deviations.

On day 1, quantifiable plasma concentrations were detected in one patient after 2 h and in all patients after 24 h. MOB015B plasma concentration increased continuously from 0.5 to 24 h up to the administration of the second dose reaching a mean C_max_ of 0.13 ng/mL and mean AUC_0-24_ of 1.55 h*ng/mL. On day 14, the mean trough concentration was 0.43 ng/mL. At the end of the treatment period (day 28), the mean trough concentration was 0.49 ng/mL, which is a 13.22% increase from day 14. The plasma MOB015B concentration–time curves are shown in Fig. 1. MOB015B concentrations steadily increased but remained at low levels on day 1 for the entire 24-h period (Fig. 1a). On day 28, there was a slight rise in concentration between 2 and 10 h (Fig. 1b), which was followed by a decline in the next 2 days to 72 h (Fig. 1c).

A summary of PK parameters is shown in Table 2. A high degree of patient-to-patient variability (coefficient of variation [CV]: >50%) was observed. On day 1, the mean T_max_ was 23.28 h, indicating a slow systemic absorption rate; in all but one patient, T_max_ was observed at the last sampling time point before the next dose on day 2. This has led to similar C_max_ and C_24_ values on day 1. A similar slow absorption is seen after multiple doses were applied, the median T_max_ on day 28 was 8.0 h (range, 0–72 h) with a 5.6-fold increase in C_max_ value compared with day 1 (0.13 to 0.72 ng/mL). Compared with day 1, a 4.35-fold increase was observed for the mean C_24_ value (0.12 to 0.53 ng/mL) and an 8.79-fold increase for the mean AUC_0-24_ value (1.55 to 13.65 h*ng/mL) on day 28.

**TABLE 2:** Summary of plasma terbinafine pharmacokinetic variables in patients treated with topical terbinafine hydrochloride 10% (MOB015B)

Variable	Time point	
	Day 1^*a*^	Day 28^*a*^
C_max_ (ng/mL)		
N^*b*^	19	19
Mean (SD)	0.13 (0.12)	0.72 (0.42)
Geometric mean	0.093	0.58
Minimum, maximum	0.022, 0.51	0.12, 1.72
C_24_ (ng/mL)		
N^*b*^	19	19
Mean (SD)	0.12 (0.11)	0.53 (0.28)
Geometric mean	0.091	0.44
Minimum, maximum	0.022, 0.51	0.10, 1.35
T_max_ (h)		
N^*b*^	19	19
Mean (SD)	23.28 (3.22)	17.90 (20.77)
Minimum, maximum	10.00, 24.40	0.00, 72.00
AUC_0-24_ (h^*a*^ng/mL)		
N^*b*^	17	19
Mean (SD)	1.55 (1.59)	13.65 (7.54)
Geometric mean	1.11	11.13
Minimum, maximum	0.44, 5.94	2.57, 29.18
AUC_0-t_ (h^*a*^ng/mL)		
N^*b*^	19	19
Mean (SD)	1.41 (1.55)	36.22 (18.80)
Geometric mean	0.94	30.43
Minimum, maximum	0.15, 5.94	6.64, 77.84

^*a*^ Results below the lower limit of quantification (10 pg/mL) were imputed as 0 before the first quantifiable concentration and imputed as missing after the first quantifiable concentration.

^*b*^ One patient withdrew from the study on day 1 and was excluded from PK analysis; in two patients, AUC_0-24_ could not be calculated.

A steady-state plasma concentration appears to have been reached by the end of dosing (day 28). The day 14 mean trough concentration, day 28 mean trough concentration and the day 28 mean C_24_ value were 0.43, 0.49, and 0.53 ng/mL, respectively. From days 14 to 28, the difference in the mean trough concentration was 0.057 ng/mL. On day 28, the difference between the mean trough concentration and mean C_24_ was 0.040 ng/mL. A minimal difference was noted between the mean C_max_ observed over the 24-h period at day 28 (0.72 ± 0.42 ng/mL, Table 2) and the mean C_max_ observed at 8 h on day 28 (0.64 ± 0.4 ng/mL, Fig. 1c).

**Fig 2:**
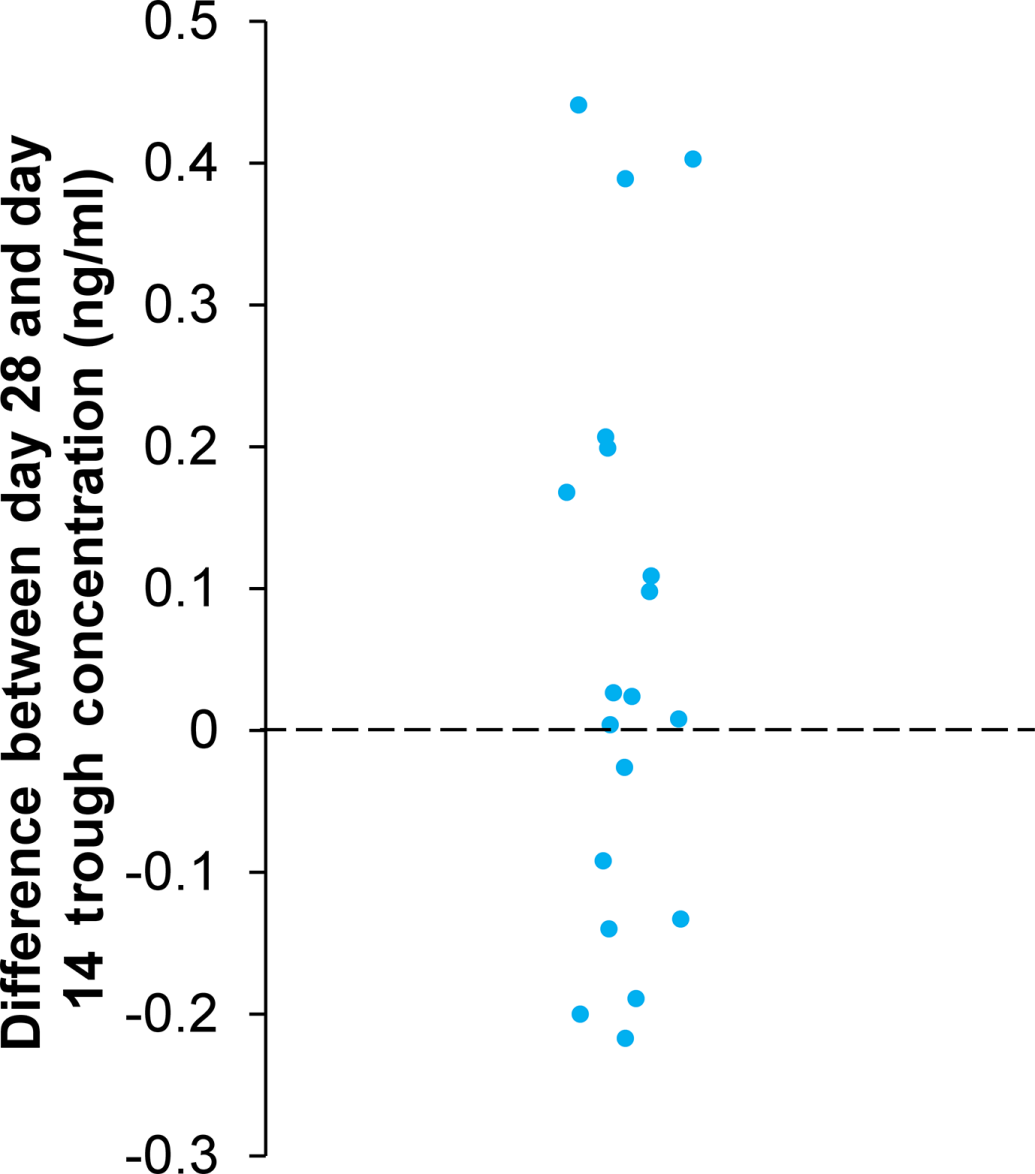
Difference between days 28 and 14 trough concentrations (*N* = 19). Compared with day 14, day 28 trough concentration increased in eight patients (0.098–0.44 ng/mL), decreased in six patients (−0.092 to −0.22 ng/mL), and exhibited minimal change in five patients (−0.026 to 0.027 ng/mL).

Owing to the patient-to-patient variabilities at the steady state, where the rate of drug absorption and the rate of drug elimination reach equilibrium, individual differences in the day 28 trough concentration compared with day 14 are presented in Fig. 2. The median change in trough concentration was minimal (median: 0.024 ng/mL [interquartile range: −0.11, 0.18]), which likely reflects the maintenance of low levels of MOB015B in plasma throughout the dosing period.

There were no local application site reactions or study discontinuations due to AEs (Table 3). Five patients (25.0%, 5/20) reported moderate treatment-emergent AE (TEAEs), including headache (*n* = 3), seasonal allergy (*n* = 1), and neck pain (*n* = 1). No patient experienced >1 TEAEs. No serious AEs were observed. All urine pregnancy tests were negative for female patients of child-bearing potential. No safety signals or clinically significant changes in vital sign measurements were observed.

**TABLE 3:** Summary of adverse events in the safety population (*N* = 20)^*b*^

Adverse event^*a*^	Value
Any AE, % (n)	25.0 (5)
Serious AEs, % (n)	0
TEAEs, % (n)	25.0 (5)
Individual TEAEs, % (n)	
Headache	15.0 (3)
Seasonal allergy	5.0 (1)
Neck pain	5.0 (1)
AEs leading to study discontinuation, % (n)	0

^*a*^ Adverse events were classified based on the MedDRA (Medical Dictionary for Regulatory Activities).

^*b*^ TEAE, treatment-emergent adverse event.

## DISCUSSION

As expected, all patients had low systemic exposure after 28 days of once-daily application of MOB015B on all toenails (day 28, mean [SD]; C_max_ = 0.72 ng/mL [0.42], AUC_0-24_ = 13.65 h*ng/mL [7.54]). These results were consistent with a previous study of MOB015B where, after 28 days of treatment, the mean plasma MOB015B concentration was 0.72 ng/mL (95% CI: 0.38, 1.05 ng/mL; median: 0.66 ng/mL [95% CI: 0.38, 1.52 ng/mL]; *n* = 8) in patients with distal subungual onychomycosis (18).

MOB015B was absorbed slowly from the toenail application site, with a further decline after the last dose was administered, consistent with low systemic absorption driven by MOB015B application to the nail plate. The maximum plasma level observed was in subject 01–007 on day 28 at 8 h (1.72 ng/mL). This is approximately 800 times lower than the mean plasma level (1.39 µg/mL) seen after oral administration of 250 mg terbinafine for 28 days (7), supporting evidence for negligible systemic absorption.

Steady-state plasma concentration appears to be reached after 28 consecutive days of treatment. The mean C_max_ observed at this point (0.72 ng/mL) is 1930.56-fold lower compared with oral terbinafine 250 mg/day after 28 days of dosing (7), indicating negligible systemic exposure. The C_max_ is also consistent with the low steady-state AUC_0-24_ (13.65 h*ng/mL), which is 835.16-fold lower than AUC reported for a single 250 mg oral dose of terbinafine (steady state AUC value of 11.4 h* µg/mL) (4). The variability on a patient-by-patient basis is expected because each patient’s metabolism and clearance of MOB015B is influenced by the individual’s state of “dynamic equilibrium.” However, we recognize that in the real-world, topical antifungals are typically applied over longer periods beyond 28 days.

Topically applied products are expected to have lower systemic exposure. Although the lack of systemic absorption especially in conditions, such as onychomycosis, is a great advantage of a topical product, such as MOB015B, the ability to penetrate the nail plate and reach the nail bed where the infection resides is key for successful clinical outcome. MOB015B concentration in the nail plate has been previously reported in eight patients after 24 weeks of daily treatment (18). The median concentration of MOB015B in the nail plate was 1,610 µg/g (mean, 3,238 µg/g). Thus, the median concentration of terbinafine in the nail plate obtained by daily application of a 10% MOB015B solution for 24 weeks is at least 1,500 times higher than the concentrations determined in nail plate tissue after 6 and 12 weeks of 250-mg oral terbinafine treatment (0.5–1.0 μg/g) (20). The potency and ability of MOB015B to cross the nail plate makes it an ideal candidate for topical therapy to treat onychomycosis.

Although male or female patients 12 to 75 years of age were allowed to participate, 85% of the enrolled patients were male, with the youngest patient being a 30-year-old male. This is due to the higher propensity for contracting onychomycosis among male patients, and among older adults as opposed to children. In a previous epidemiological survey, males exhibited 2.4-fold higher odds for contracting onychomycosis than females (21); this difference in propensity between the sexes is also reflected in phase three onychomycosis trials (16, 22). The main risk factors in the elderly are repeated trauma, immunosuppression, slower rate of nail growth, and decreased peripheral circulation (23). In children following a single weight-adjusted dose of oral terbinafine (125 mg), the time to reach maximum plasma concentration (T_max_) was similar to adults indicating a comparable rate of absorption (mean T_max_ in children: 1.7–2.1 h, mean T_max_ in adults: 1.4–1.5 h) (24, 25), with a lower degree of absorption after adjusting for body weight (mean C_max_ in children: 167 ng/mL, mean C_max_ in adult: 357 ng/mL) (24).

The therapeutic utility of topical terbinafine lies in its improved safety profile, thereby favoring its use as a long-term treatment option with no risk of clinically significant systemic exposure, especially given the long-term treatment required due to the slow rate of toenail growth (26). This formulation is also recommended as a prophylaxis to reduce the risk of recurrence (12, 13), as well as in patients for whom oral terbinafine is contraindicated (27, 28).

In this study, terbinafine hydrochloride 10% solution (MOB015B)—administered under maximal use conditions—was found to be safe and well-tolerated with no reported severe TEAEs, serious AEs, or AEs leading to study discontinuation. The plasma terbinafine levels in MOB015B treated patients were significantly lower than those of oral terbinafine indicating a lower systemic exposure risk.

This observation supports earlier findings that MOB015B accumulates primarily in the nail plate, thereby allowing its release into the nail bed (18). The low level of systemic exposure is also reflected by the favorable safety and tolerability profile observed during this study. Some limitations of our study include the relatively short treatment duration of 28 days, and the lack of measurement of MOB015B levels in the nail bed and nail plate. Further clinical validation is warranted to optimize its use in onychomycosis management.

## MATERIALS AND METHODS

### Study design and enrollment

**Fig 3:**
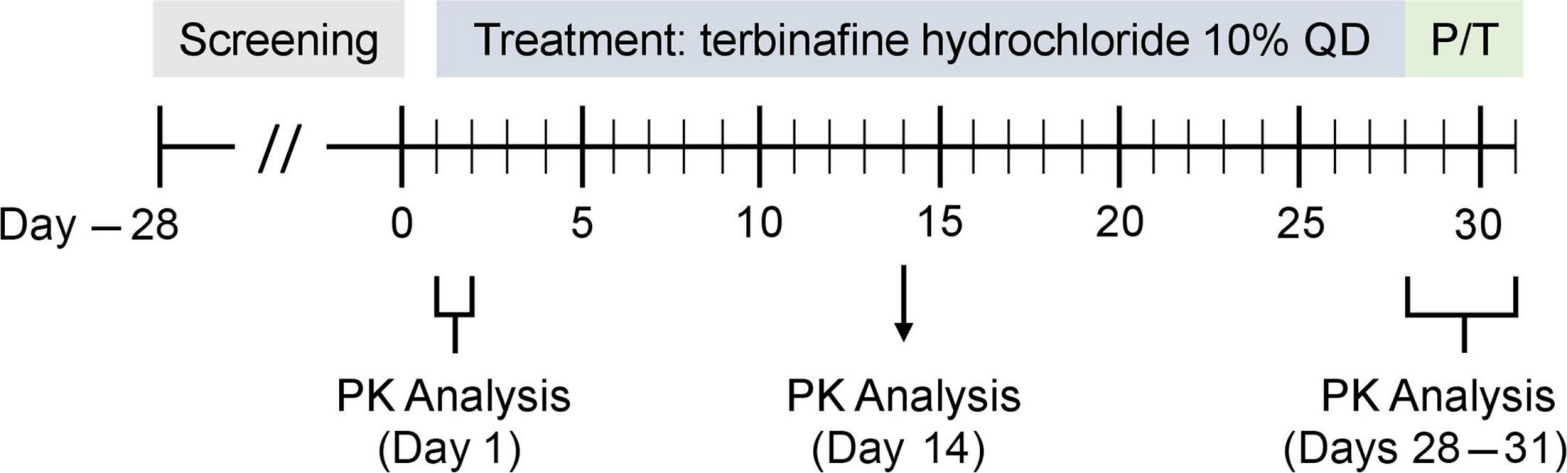
Study design. P/T, post-treatment; QD, once daily.

This was a 31-day, single-center, open-label dosing trial of adult patients with onychomycosis treated with MOB015B, a terbinafine hydrochloride 10% solution, under maximal use conditions (Fig. 3). Institutional Review Board approval was obtained for the study protocol and informed consent form (Schulman Associates IRB, Inc.). The study was prospectively registered (ClinicalTrials.gov, NCT03244280), and conducted in accordance with the Declaration of Helsinki and globally accepted standards of good clinical practice. The study adhered to local regulations. All patients provided written informed consent. The amount of drug used per application, the patient characteristics, and the 28-day duration of treatment are industry standards used for establishing the pharmacokinetics and systemic absorption of topical onychomycosis products.

Patients aged between 12 and 75 years with moderate-to-severe subungual toenail onychomycosis, indicated by ≥50% involvement of both halluces and additional involvements of least four other toenails, were enrolled. In addition to clinical diagnosis, patients must have had a positive direct KOH microscopy confirmation of at least one hallux at screening, and a body mass index of 18.5–35.0 kg/m^2^. Female individuals of child-bearing potential were excluded if they were pregnant or planning to become pregnant. Patients who had been treated with oral terbinafine within 12 months or topical terbinafine within 6 months were excluded.

Patients received instructions to apply MOB015B once daily to each toenail for 28 days consecutively. The study drug was to be applied on the nail plate dorsum as a thin layer, as well as under the distal free edge and hyponychium. Each application was carried out in the morning and allowed to dry for 5 min; patients were instructed not to wash their feet for at least 8 h after each application. Use of cosmetic nail products was prohibited throughout the study period.

### Drug description

The study drug was provided in a 10% hydrochloride solution corresponding to 8.9% terbinafine formulated in urea, propylene, and lactic acid. The solution was supplied in 10mL polyethylene plastic tubes with a silicon tip to facilitate topical applications.

### Outcome measures

To evaluate systemic absorption, all patients underwent blood draws to determine the plasma MOB015B concentrations on days 1 (baseline), 14, and 28 (end of treatment). On day 1, patients applied MOB015B to each toenail after a pre-treatment blood sample was taken (0 h), and treatment continued daily until day 28 (end of treatment). On day 1, additional samples were collected at 0.5, 1, 2, 4, 6, 8, 10, and 24 h post-dose. A pre-dose sample was collected on days 14 and 28. On day 28, additional samples were collected at 0.5, 1, 2, 4, 6, 8, 10, 24, 48, and 72 h post-dose. Subsequently, 2- to 3-mL blood samples from each patient were collected in a K2 EDTA tube at the Clinical Research Site and were shipped frozen on dry ice to the bioanalytical laboratory. The dates of receipt and internal tracking numbers were assigned to each shipment and filed for laboratory record keeping. The study samples were stored in a freezer at the bioanalytical laboratory at a nominal temperature of −70°C ± 10°C before the analysis.

A liquid chromatography–mass spectrometry method for human plasma in K_2_EDTA was utilized with a predetermined detection range of 10–5,000 pg/mL for terbinafine (Frontage Laboratories, Inc., Exton, PA, USA); terbinafine-d_7_ HCl was used as the internal control. Liquid chromatography was conducted using a C18 column (Agilent, Zorbax, Eclipse Plus C18) with appropriate mobile phases. Intra-run and inter-run accuracy and precision were tested against three quality control concentrations (30, 750, and 3750 pg/mL), the overall accuracy was within ±15% of the nominal value with CV ≤15%. Stability testing demonstrated that terbinafine was stable in human K_2_EDTA plasma for 16.5 h at room temperature, up to 35 days at −20°C and −70°C, and after three freeze/thaw cycles at −20°C and −70°C.

Evaluated PK parameters included C_max_ (maximum plasma concentration), C_24_ (plasma concentration at 24 h after dosing), T_max_ (time to reach C_max_), AUC_0-24_ (area under the plasma concentration–time curve from 0 to 24 h after dosing), and AUC_0-t_ (area under the plasma concentration–time curve from 0 h to the time of the last quantifiable plasma concentration).

AEs were reported based on the preferred terms of the Medical Dictionary of Regulatory Activities and categorized based on the system organ class. AE severity was coded as mild (awareness of sign or symptoms, but easily tolerated), moderate (moderate discomfort enough to cause interference with usual activity), or severe (severe incapacitation with inability to work or perform usual activity). Serious AEs, including death, a life-threatening event, requiring inpatient hospitalization or causing prolonged hospitalization, were also recorded. Possible causative relationships between the study drug and safety events were categorized into five groups from not related to definitely related. Vital signs were measured at screening, days 1, 14, and 28. Urine pregnancy tests were conducted on days 1, 14, and 31. Screening for alcohol use or drug use was conducted at screening and on day 1.

### Data analysis

The PK population included all patients who received the study drug with evaluable concentration–time profiles. The safety population included all enrolled patients.

Analysis of PK parameters was carried out using Phoenix WinNonlin^®^ Non-Compartmental Analysis (NCA). C_max_, C_24_, and T_max_ were derived directly from experimental observations; AUC_0-24_ and AUC_0-t_ were calculated using the linear trapezoidal rule. Descriptive statistics for plasma terbinafine concentrations include the arithmetic mean, standard deviation (SD), geometric mean, CV, minimum, and maximum. For samples with concentrations below the lower limit of quantification of 10 pg/mL (0.01 ng/mL), the value was imputed as 0 for summary descriptive statistics. For NCA, concentration of samples below the lower limit of quantification was set to 0 before the first quantifiable concentration and set to missing after the first quantifiable concentration. Any quantified concentrations detected at the pre-dose time point on day 1 (baseline) were set to 0.
